# The Virginia Medical and Surgical Journal

**Published:** 1853-04

**Authors:** 


*Virginia Medical and Surgical Journal,* is a new competitor for professional patronage. It opens remarkably well. The original department is extensive and varied, and the selections evince much judgment and care. We confidently recommend it to the favorable consideration of our readers, fully satisfied that it will amply repay an attentive perusal.

